# A Rare Cause of Acute Abdomen: Perforation of Double Meckel's Diverticulum

**DOI:** 10.1155/2015/648417

**Published:** 2015-07-22

**Authors:** İlhan Tas, Serdar Culcu, Yigit Duzkoylu, Sadik Eryilmaz, Mehmet Mehdi Deniz, Deniz Yilmaz

**Affiliations:** ^1^General Surgery Clinic, Cizre Dr. Selahattin Cizrelioglu State Hospital, Şırnak, Turkey; ^2^General Surgery Clinic, Islahiye State Hospital, Gaziantep, Turkey; ^3^General Surgery Clinic, Bayrampasa State Hospital, Istanbul, Turkey; ^4^Pathology Department, Cizre Dr. Selahattin Cizrelioglu State Hospital, Şırnak, Turkey

## Abstract

Meckel's diverticulum is the most common congenital anomaly of the gastrointestinal tract. In this report, we aimed to represent a case of intestinal perforation, caused by double Meckel's diverticulum, which is a very rare entity in surgical practice. The patient was a 20-year-old Caucasian man, admitted to hospital with complaints of abdominal pain, nausea, and vomitting during the last 3 days. Physical examination indicated tenderness, rebound, and guarding in the right lower quadrant of abdomen. Abdominal X-ray revealed a few air-liquid levels in the left upper quadrant. In the operation, 2 Meckel's diverticula were observed, one at the antimesenteric side, at 70 cm distance to the ileocecal valve, approximately in 3 cm size, and the other between the mesenteric and antimesenteric sides, approximately in 5 cm size. The first one had been perforated at the tip and wrapped with omentum. A 30 cm ileal resection, including both diverticula with end-to-end anastomosis, was performed. The diagnosis of symptomatic Meckel's diverticulum is considerably hard, especially when it is complicated. Diverticulectomy or segmentary resections are therapeutic options. In patients with acute abdomen clinic, Meckel's diverticulum and its complications should be kept in mind, and the intestines should be observed for an extra diverticulum for caution although it is a very rare condition.

## 1. Introduction

Meckel's diverticulum is the most common congenital anomaly of the gastrointestinal tract, with the incidence of 1–3% [1–4]. Incomplete obliteration of the omphalomesenteric duct in intrauterine period is thought to be the main etiologic factor [1, 5]. Preoperative diagnosis is compelling especially in uncomplicated cases. Meckel's diverticulum is symptomatic in 4–6% of the patients [5, 6]. The ratio of men to women in symptomatic cases is reported to be 3/1 [2, 7]. Most common complications include ulceration, intestinal obstruction, hemorrhage, perforation, intussusception, vesicodiverticular fistula, and malignancy [3, 8, 9]. In our study, we aimed to report a rare case of double Meckel's diverticulum, resulting in intestinal perforation, mentioning the diagnosis, treatment, and postoperative results.

## 2. Case

The patient was a 20-year-old Caucasian male, admitted to the Emergency Clinic 3 days ago, diagnosed as nonspecific abdominal pain, and discharged with symptomatic treatment. Later he was admitted to General Surgery Clinic with clinical signs of acute abdomen. He had neither any comorbidities, nor any specificities in his history.

In his physical examination, blood pressure was found to be 120/70 mmHg, pulse was 86/minutes, respiration rate was 16/minutes, and the temperature was 36.8°C. There were tenderness, guarding, and rebound in the right lower quadrant of abdomen. Digital rectal examination was normal. In his laboratory findings, WBC was found to be 11.200, hemoglobin was 12.8 g/dL, and platelet count was 133.000. Abdominal X-ray revealed a few air-liquid levels in the left upper quadrant. In abdominal ultrasonography, intestinal ansae were found to be dilated at the lower quadrants, and a noncompressible appendix in 6 mm thickness was observed at the right lower quadrant.

**Figure 1:**
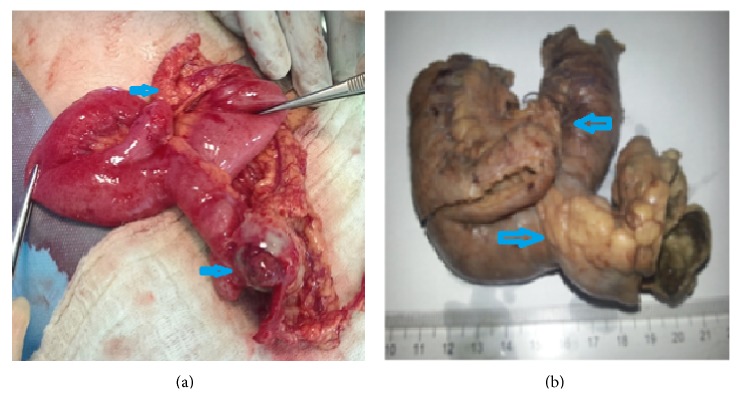
(a) Double Meckel's diverticulum, one wrapped with omentum. (b) Two diverticula, located in the resected material; the right one is inflamed.

Following adequate hydration and double antibiotic prophylaxis with 1 gr of ceftriaxone and 500 mg of metronidazole, the patient was taken to operation at emergency conditions. Following a Mc Burney incision, appendix was found to be inflamed, with extensive reactional free fluid in the cavity. After performing appendectomy, a mass was palpated in the abdomen, and intestinal ansae were taken out of the abdomen for exploration. Two diverticula were observed, one on the antimesenteric side, at 70 cm distance to the ileocecal valve, in 3 cm size, and the second one between the mesenteric and antimesenteric sides, in 5 cm size. The first diverticulum was found to be perforated at the tip and wrapped with omentum (Figure 1). An ileal resection of 30 cm, including the diverticula and end-to-end anastomosis, was performed, because the abdominal cavity was not contaminated. A drainage tube was inserted into the rectovesical pouch.

**Figure 2:**
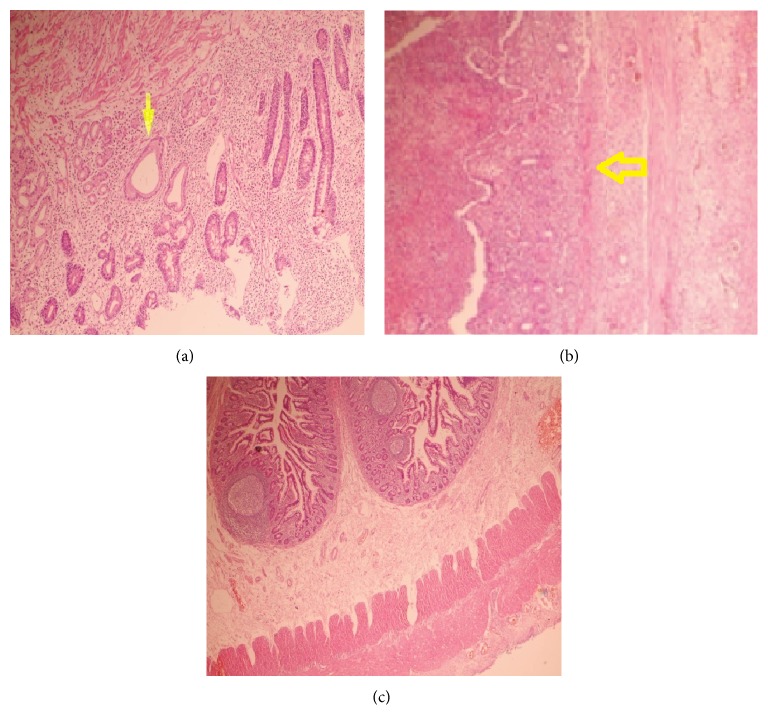
(a) Gastric mucosa (arrow), at the side of ulcerated intestinal mucosa (HE ×100). (b) Ulcerated intestinal wall (HE ×200). (c) Histopathology of the second diverticulum (HE ×200).

Gaita discharge started on the postoperative second day, and oral intake was allowed on the fourth day. The patient was discharged on postoperative sixth day without any complications. Histopathologic examination revealed perforation signs in the larger diverticulum with heterotopic gastric mucosa and active inflammation hemorrhage and edema in the smaller one (Figure 2).

## 3. Discussion

Meckel's diverticulum is the most frequent congenital anomaly of the gastrointestinal tract, resulting from the incomplete atrophy of the omphalomesenteric duct [1–3, 5, 10]. Heterotopic tissues such as gastric, duodenal, colonic ones and rarely pancreatic mucosa can be found in the diverticula, as well as the anatomically normal intestinal mucosa [9, 10]. They are found in 15–50% of the cases and more often in symptomatic patients [10]. Preoperative diagnosis is rare in uncomplicated cases, and the diverticulum is usually observed incidentally, during other procedures for various reasons [11]. “Rule of twos” is characteristic for Meckel's diverticulum, which includes the prevalence in 2% of the population; it is usually diagnosed under the age of 2; it is in 2-inches size and 2 cm diameter, 2 feets proximal to the valve, twice frequent in men, and symptomatic in 2% of the patients [11, 12].

Double Meckel's diverticulum is a rare condition, and the first study was reported by Emre et al., representing 5 cases with double diverticula [1]. In our case, the rare entity is the observation of double Meckel's diverticulum and perforation in one of them. Although preoperative diagnosis may be compelling, and the most frequently used modalities are computerized tomography (CT), Technetium-99 m pertechnetate scintigraphy, and double-balloon enteroscopy, which is superior to the others [13], scintigraphy has the capability of observing ectopic gastric mucosa but may have false positive and negative results at high rates [10].

In our case, we performed an emergent operation with the initial diagnosis of acute appendicitis, relying on the physical examination findings and Meckel's diverticulum was observed incidentally. The most useful imaging method is CT in the diagnosis of intestinal obstruction, which is a possible complication of the disease [12]. Laparoscopy may also be useful, in both diagnosis and treatment [10]. Surgery is the gold standard treatment option in symptomatic patients. Diverticulectomy or segmentary resection with anastomosis is the surgical options. Mortality rates are reported to be 1.6–7.7% [6]. Laparoscopic procedures have taken conventional operations' place in the recent years, providing two different surgical options, intracorporeal laparoscopy and TULA (transumbilical laparoscopic-assisted Meckel's diverticulectomy), which are useful in both diagnosis and treatment. The most important disadvantage of the former technique is to leave heterotopic tissue at the distal parts, especially when it is performed with staplers [10].

## 4. Conclusion

In patients with clinical signs of acute abdomen, Meckel's diverticulum and its potential complications should be kept in mind, especially when any reasons that can explain the clinical findings of the patient are not observed intraoperatively.
